# Comparative analysis of the organelle genomes of seven *Rosa* species (Rosaceae): insights into structural variation and phylogenetic position

**DOI:** 10.3389/fpls.2025.1584289

**Published:** 2025-05-08

**Authors:** Rongxiang Zhang, Shuwen Liu, Ying Liu, Pei Wei, Niyan Xiang, Yuemei Zhao, Xiaoman Gao, Yebing Yin, Lijun Qin, Tao Yuan

**Affiliations:** ^1^ School of Biological Science, Guizhou Education University, Guiyang, China; ^2^ State Key Laboratory of Hybrid Rice, Laboratory of Plant Systematics and Evolutionary Biology, College of Life Sciences, Wuhan University, Wuhan, China; ^3^ School of Resources and Environmental Science, Hubei University, Wuhan, China; ^4^ School of Ecology and Environment, Tibet University, Lhasa, China; ^5^ Key Laboratory of Plant Resource Conservation and Germplasm Innovation in Mountainous Region (Ministry of Education), College of Life Sciences/Institute of Agro-bioengineering, Guizhou University, Guiyang, Guizhou, China

**Keywords:** *Rosa*, organelle, phylogeny, structural, adaptation

## Abstract

**Introduction:**

The genus *Rosa* belongs to the family Rosaceae within the order Rosales, which is one of the more ancient plant lineages. At present, the complete mitochondrial genome of *Rosa* spp. is still rarely reported, and studies on the mitochondrial genome of Rosa spp. are limited.

**Methods:**

In this study, the *R. laevigata* mitochondrial genome was sequenced using both Pacbio Sequel II and DNB-SEQ-T7 platforms. The second- and third-generation data for the other five Rosa species were downloaded from the NCBI database. Genome annotation was performed using Geneious, with structural visualization via CPGview. In-depth analyses were conducted, including assessments of non-synonymous/synonymous mutation ratios (Ka/Ks), codon usage bias, collinearity, and the identification of homologous fragments between chloroplast and mitochondrial genomes. Finally, we employed the maximum likelihood (ML) methods to analyze the phylogenetic relationships among *R. laevigata* and other *Rosa* species.

**Results:**

The chloroplast genome sizes ranged from 156,342 bp (*R. laevigata*) to 157,214 bp (*R. agrestis*). The GC content varied from 37.2% to 37.3%, and the number of genes ranged from 129 to 131. The mitochondrial genomes were all circular, with lengths between 271,191 bp and 338,975 bp, containing 52 to 59 genes. Codon usage analysis indicated a preference for A/T-ending codons in both chloroplast and mitochondrial genes. Four highly differentiated regions (*rps19*, *ndhF*, *ycf1*, and *psbM-trnD-GUC*) in the plastomes of the 7 *Rosa* species were identified, which can serve as molecular markers for future species identification and studies of genetic diversity. Compared to PCGs of plastome, mitochondrial PCGs displayed a higher non-synonymous to synonymous ratio. We also observed extensive gene transfer between the mitochondria and chloroplasts, particularly with the rrn16 and *rpl23* genes, which are commonly found in *Rosa* species. These gene transfer events likely occurred in the ancestor of *Rosa* around 4.46 Mya. Estimates of divergence events indicate that rapid differentiation among *Rosa* species took place around 4.46 Mya, potentially influenced by the uplift of the Qinghai-Tibet Plateau during the Late Miocene.

**Discussion:**

This study enriches the genetic resources of the *Rosa* genus and lays the groundwork for the development of molecular markers, phylogenetic analyses, and research into the evolution of organelle genomes.

## 1 Introduction

The genus *Rosa* belongs to the family Rosaceae within the order *Rosa*les, which is one of the more ancient plant lineages. *Rosa* species are semi-woody perennials that are cultivated globally as ornamental plants (Gahlaut et al., 2021). With a diverse array of species and colors, *Rosa* species are among the most diverse horticultural crops. They have extensive applications in landscaping, home gardening, perfumery, cosmetics, and pharmaceuticals, offering substantial economic, aesthetic, and medicinal value to both people’s lives and industries. Currently, approximately 200 species of the *Rosa* genus are recognized worldwide, distributed mainly in cold-temperate to subtropical regions, including Asia, Europe, North America, and North Africa. In China, there are 95 species, 65 of which are endemic, such as *Rosa* taronensis, *R*. *omeiensis*, and *R*. *longicuspis* (Wu and Chen, 1974; Lu et al., 2003; Joichi et al., 2005; Potter et al., 2007; Fougere-Danezan et al., 2015). The classification of the *Rosa* genus can be challenging due to blurred species boundaries and complex interspecific relationships, which are often further complicated by trait similarities resulting from artificial breeding. Consequently, Focke’s subgenus concept is currently applied in China to divide the *Rosa* genus into two subgenera: Subgen. Rosa and Subgen. Hulthemia, which includes 7 lineages and 9 groups (Wu and Chen, 1974; Lu et al., 2003; Joichi et al., 2005; Bruneau et al., 2007). Issues such as unclear species delimitation, low sequence variation, and incomplete lineage sorting (ILS) have posed significant challenges to the molecular taxonomy of *Rosa* species. Advances in sequencing technology have made integrated molecular taxonomic studies utilizing plastomes, ribosomal genes, and nuclear genome fragments increasingly mainstream in plant taxonomy (Zhu and Gao, 2015; Zhang et al., 2020). Although the understanding of the *Rosa* genus dates back quite some time, several factors present significant challenges to its classification. The *Rosa* genus is widely distributed, with many species capable of hybridizing, which has led to multiple instances of chromosomal duplication (Zong et al., 2024). This contributes to numerous phenotypic similarities among species, further complicated by human interventions such as hybrid breeding and transcontinental migrations. As a result, the genus includes similar traits, along with challenges like multiple synonyms for the same species and inconsistent phenotypic expressions across different regions. To date, several researchers have attempted to reconstruct the phylogenetic relationships within the *Rosa* genus. However, integrating molecular taxonomy, cytological taxonomy, and classical taxonomy has not yielded satisfactory results. Many of the traits used for characterization in the *Rosa* genus appear remarkably similar, yet significant differences emerge at the cellular and molecular levels, making it challenging to consolidate them into a cohesive phylogenetic framework. Thus, the phylogenetic relationships within the *Rosa* genus remain a pressing issue in plant taxonomy.

However, organelle genomes still hold significant application in these analysis mentioned above. These genomes play a crucial role in species identification and the reconstruction of evolutionary histories, which conserved genetic features and low levels of gene flow (Smith and Keeling, ,P, 2015; Wang et al., 2024b). Meanwhile, due to the limited availability of genomic data, organelle genomes remain the predominant method for phylogenetic studies of *Rosa* species. The plastome is an organelle with a semi-autonomous genetic system that exists independently of the nuclear genome. Since the first plastome from the genus *Rosa* was reported in 2014 (Yang et al., 2014), the NCBI has cataloged 51 complete plastomes from various *Rosa* species. Phylogenetic studies based on these plastomes indicate that the phylogenetic relationships among *Rosa* species are generally consistent with current taxonomic classifications (Engler System) (Niu et al., 2022; Shen et al., 2022). However, many *Rosa* species remain unrepresented, Highlighting the need for further sequencing of unreported species to enhance our understanding of phylogenetic relationships within the genus *Rosa*. The mitogenome also features an independent semi-autonomous genetic system separate from the nuclear genome. However, unlike plastomes, mitogenomes in higher plants display substantial size variation and structural complexity, ranging from a few kb to several Mb, and can be linear, circular, or multi-circular (Guo et al., 2023; Zhang et al., 2024). Although mitogenome sequencing has been completed for many plant species (Wu et al., 2020; Bi et al., 2023; Wang et al., 2023), research on the mitogenomes of *Rosa* species is quite limited. The mitogenome of *R. chinensis* was first reported by Raymond et al (Raymond et al., 2018), followed by the report of *R. angusta*’s mitogenome by Park et al. in 2020 (Park et al., 2020). In this study, we performed PacBio HiFi sequencing of *R. laevigata*, along with the assembly and annotation of their mitochondrial genomes in conjunction with 5 other *Rosa* species (*R. agrestis, R. canina*, R. r*oxburghii*, R. sp*inosissima*, and R. *sterilis*) for which third-generation sequencing data are currently available. Prior to this study, only 3 mitogenomes (R. rugosa, R. chinensis, R. hybrid) were available for the entire *Rosa* genus. Our findings substantially enrich this genomic resource by adding multiple new mitochondrial sequences.

Our study annotated and assembled the mitogenomes and plastomes of 6 previously unreported *Rosa* species, thereby enriching the *Rosa* organellar genome database. Additionally, we conducted intergeneric comparative analyses of the reported plastomes to investigate genomic characteristics, evolutionary relationships, and phylogenetic connections, providing a foundational basis for future research on *Rosa* organellar genomes. This study aims to assemble the plastomes and mitogenomes of previously unreported *Rosa* species. By combining these new sequences with existing plastomes data, we seek to reconstruct phylogenetic relationships within the genus, explore the connections and classification boundaries among various species groups, and identify regions of genetic variability. The findings from this research will provide valuable insights into the development of molecular markers for the *Rosa* species, as well as for phylogenetic studies and taxonomic revisions.

## 2 Results

### 2.1 Characterization of organelle genes

**Figure 1:**
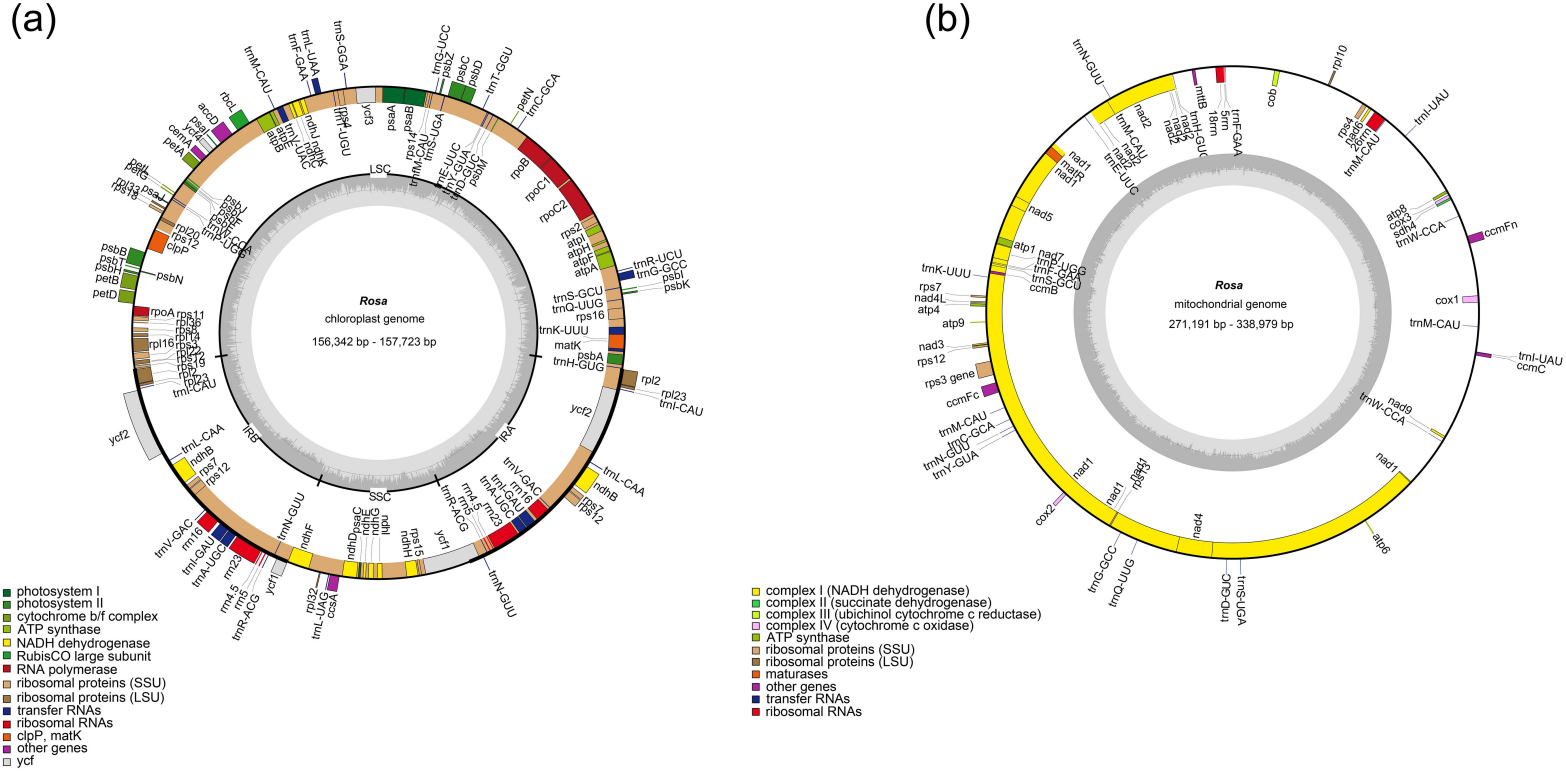
Genome map of **(a)** the plastome and **(b)** mitogenome of *Rosa* species. Genes belonging to functional groups are color-coded on the circle as transcribed clockwise (outside) and transcribed counterclockwise (inside). The darker grey in the inner circle represents the GC content, while the lighter grey represents the AT content.

The complete plastomes of the 7 *Rosa* species ranged in length from 156,342 bp (*R. laevigata*) to 157,214 bp (*R. agrestis*), with a maximum difference of 872 bp and a minimum difference of 43 bp (**Figure 1**; **Table 1**). The GC content varied between 37.2% and 37.3%. Minor differences were observed in the number of genes across species, which ranged from 129 to 131, as shown in **Table 1**. The mitogenomes of all 7 *Rosa* species were circular in structure, with lengths ranging from 271,191 bp to 338,979 bp and GC contents between 45.0% and 45.6% (**Figure 1**; **Table 2**). Differences in the number of mitochondrial genes among species ranged from 52 to 59. Our results indicated that the mitogenomes exhibited greater fluctuations in both GC content and genome size. By contrast, the plastomes showed more conserved GC content and less variation in genome size throughout evolution.

**Table 1:** Plastome characteristics of 7 *Rosa* species.

Species	Genome size (bp)	Number of genes	Protein genes	tRNA genes	rRNA genes	GC contents (%)
*R. agrestis*	157,214	131	81	38	8	37.3
*R. canina*	156,589	129	81	37	8	37.2
*R. laevigata*	156,342	139	87	39	8	37.3
*R. roxburghii*	156,723	130	81	37	8	37.2
*R. spinosissima*	157,171	130	81	37	8	37.2
*R. sterilis*	156,561	130	81	37	8	37.2
*R. rugosa*	157,120	130	84	37	8	37.2

**Table 2:** Mitogenome characteristics of 7 *Rosa* species.

Species	Genome size (bp)	Number of genes	Protein genes	tRNA genes	rRNA genes	GC contents (%)
*R. agrestis*	271,192	54	31	20	3	45.4
*R. canina*	273,201	59	32	24	3	45.6
*R. laevigata*	282,374	54	31	20	3	45.4
*R. roxburghii*	323,977	57	30	24	3	45.4
*R. spinosissima*	290,804	53	30	20	3	45.2
*R. sterilis*	338,979	54	30	23	3	45.0
*R. rugosa*	302,947	54	31	20	3	45.2

### 2.2 Comparative analysis of plastomes

The overall sequence identity analysis of the plastomes of 7 species within the genus *Rosa* was conducted using mVISTA, with *R. rugosa* as the reference. The results indicate that the plastomes of these 7 *Rosa* species exhibit a high degree of sequence similarity, with the inverted repeat (IR) regions being more conserved than the single-copy regions (**Supplementary Figure S1**). Genetic variation is lower in the coding regions than in the non-coding regions. Three highly variable regions were identified in the coding sequences: *rps19*, *ndhF*, and *ycf1*. Additionally, a highly variable region was observed in the non-coding region (*psbM*-*trnD*-*GUC*) (**Supplementary Figure S1**). The IR regions, being the most conserved areas of the chloroplast genome, can undergo expansions and contractions at their boundaries, which can result in changes to the overall genome length (Zhang et al., 2024).

**Figure 2:**
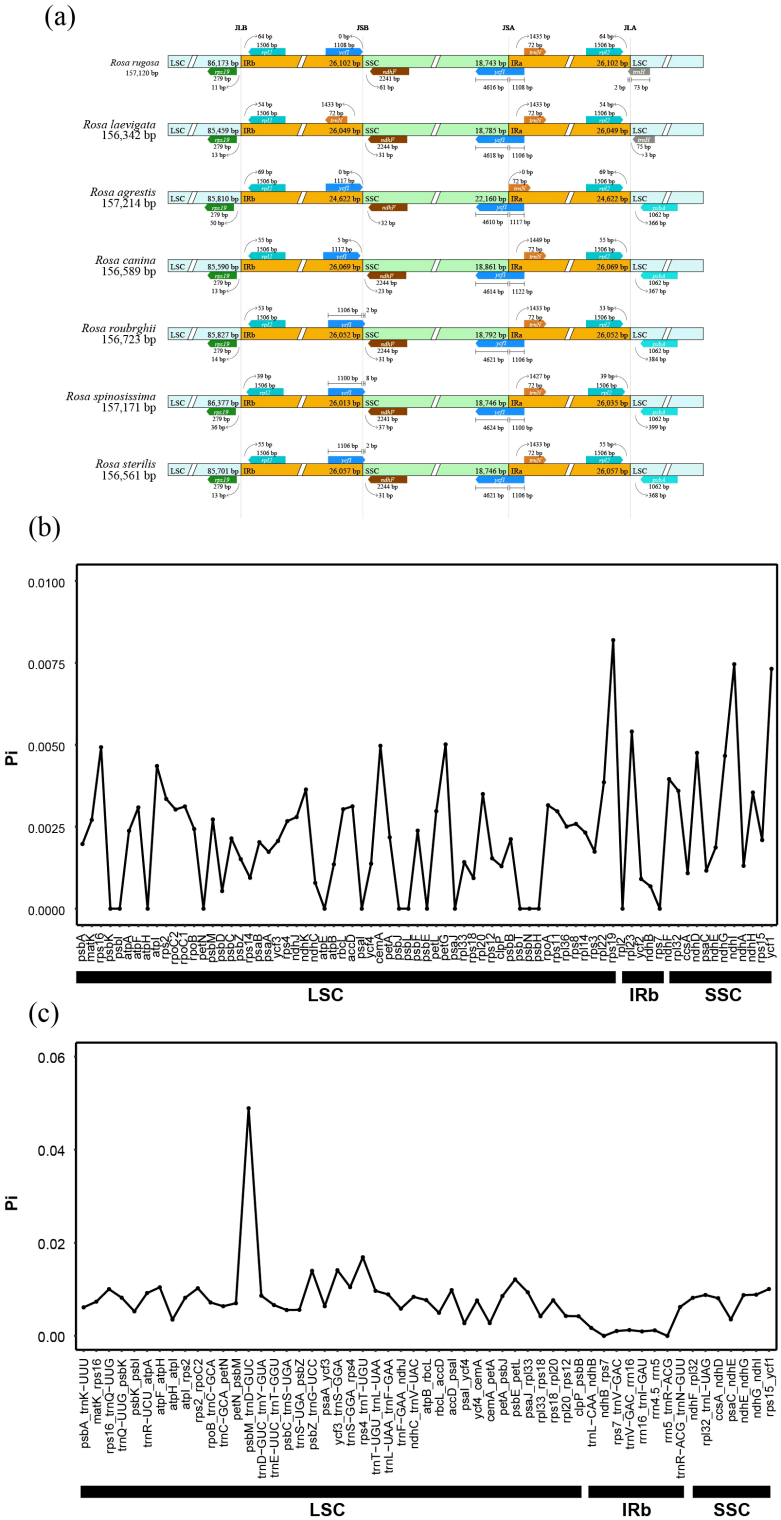
Comparative plastome analysis. **(a)** Comparison of borders between LSC, SSC and IRs regions across the plastomes of *Rosa* species. **(b)** Sliding-window analysis of gene coding regions in plastomes for 7 *Rosa* species. **(c)** Sliding-window analysis of non-coding regions in plastomes for 7 *Rosa* species. X-axis: position of the midpoint of a window; Y-axis: nucleotide diversity (Pi) of each window.

This study conducted a comparative analysis of the IR/SSC boundary regions among the plastomes of the 7 *Rosa* species (**Figure 2**). We found subtle differences at the IR/SSC boundaries across the 7 species. The IRb/LSC (JLB) junctionlies in the intergenic region between the *rps19* and *rpl2* genes, while the SSC/IRa (JSA) junction is situated on the *ycf1* gene. The difference of regions primarily occur at IRb/SSC (JSB) and IRa/LSC (JLA), with *R. rubiginosa*, *R.* sp*inosissima*, and *R. sterilis* showing IR contraction into the *ycf1* gene at JSB. The JLA for *R. rugosa* and *R. laevigata* is located between the *rpl2* and *trnH* genes, whereas for the other species, it is positioned between *rpl2* and *psbA*.

Furthermore, an analysis of nucleotide polymorphism (Pi) based on 78 plastome coding and non-coding regions across the 7 *Rosa* species revealed notable patterns. Among the 78 PCGs, *rps19* exhibited the highest Pi value (0.009182524), followed by *ndhI* (0.007458405) and *ycf1*(0.007316403) (**Figure 2**). In the non-coding regions, the highest Pi value was observed in psbM_trnD-GUC (0.048908538), followed by *rps4_trnT-UGU* and *ycf3_trnS-GGA*, with Pi values of 0.016897081 and 0.014111365, respectively (**Figure 2**). These findings suggested that the Pi values are significantly higher in the non-coding regions compared to coding regions. Additionally, the elevated Pi values in the PCGs, particularly in *ndhI* and *ycf1*, indicated a relatively high level of nucleotide polymorphism and highlighted their potential as genetic markers for phylogenetic studies.

### 2.3 Codon usage and ENc-GC3s analysis

**Figure 3:**
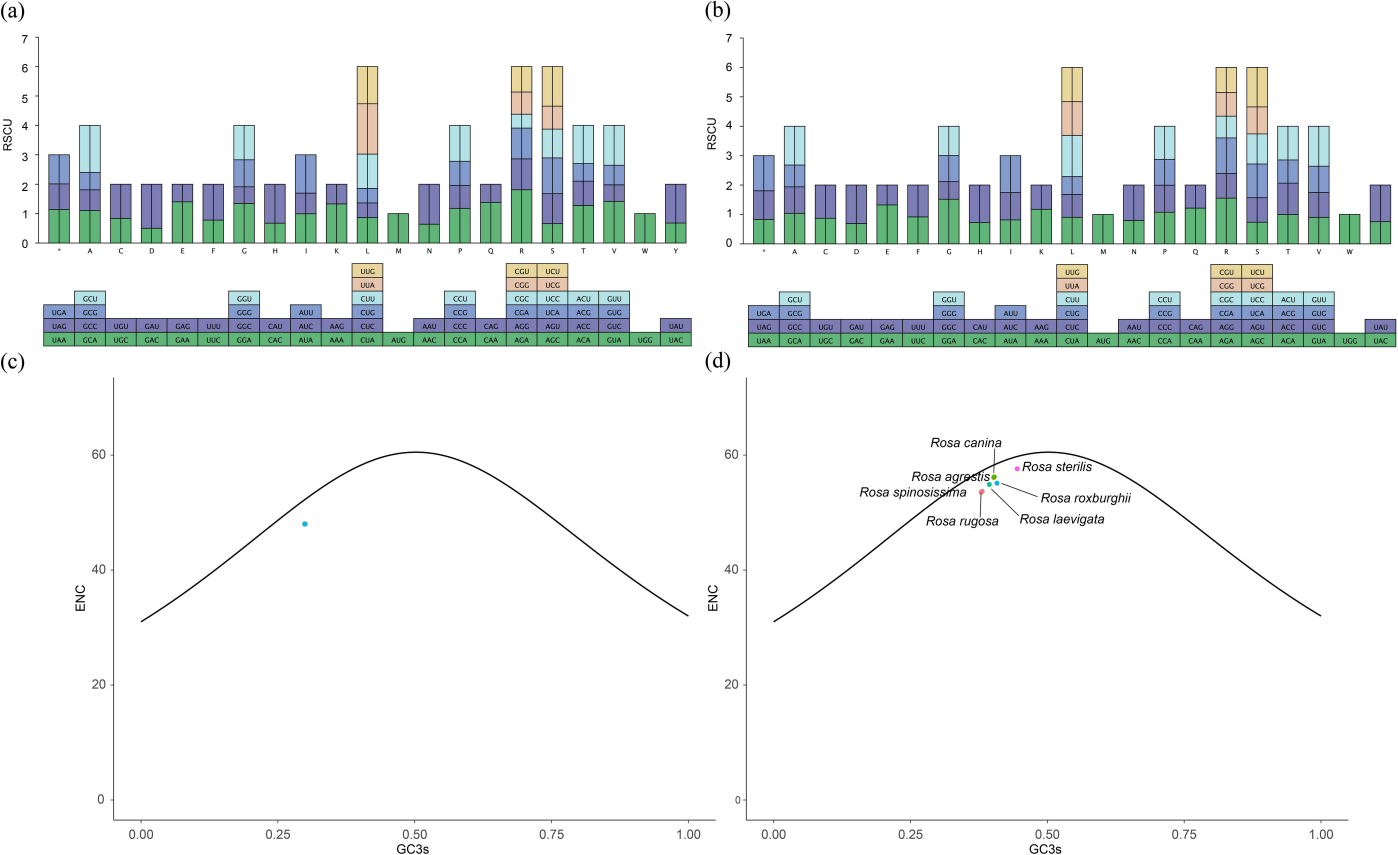
Relative synonymous codon usage (RSCU) and ENC plotted against GC3s based on PCGs of 7 *Rosa* organelle genes. **(a)** the plastome genes. **(b)** the mitogenome gene. **(c)** the plastome PCGs. **(d)** the mitogenome PCGs. The solid line indicates the expected curve of positions of genes when the codon usage is merely determined by the GC3s composition.

The codon usage of protein-coding genes in the chloroplasts and mitochondria of 7 species of the *Rosa* genus is illustrated in **Figure 3**. Phenylalanine (Phe) is the most frequently used amino acid among these organelles, accounting for 3.89% in chloroplast and 6.63% in mitochondria, respectively (**Supplementary Table 3**). Following Phe, isoleucine (Ile) ranks next, comprising 3.84% and 2.96%. This appears to be a common phenomenon, as similar findings have been observed in the organellar genomes of *Aconitum* (Xia et al., 2022; Zhang et al., 2024). Additionally, we performed a statistical analysis of codon usage in the PCGs of the plastomes and mitogenomes across the 7 species. In the plastome, there are 31 codons with RSCU values greater than 1, 31 less than 1, and 2 equal to 1. Among these, AGA has the highest RSCU value (1.8114), while CGC has the lowest (0.4725). Of the codons with RSCU values greater than 1, 13 end with A, 15 with U, and 1 with G; none end with C. A similar pattern is observed in the mitogenome, where 27 codons have RSCU values greater than 1, 35 less than 1, and 2 equal to 1. Again, AGA has the highest RSCU value (1.5553) and CGC has the lowest (0.5963). Among these, 11 end with A, 13 with U, 2 with C, and none with G. These findings suggested a general trend, as reported in *Aconitum* species (Meng et al., 2018; Zhang et al., 2024).

To explore the relationship between nucleotide composition and codon usage, we analyzed the GC content and codon usage of PCGs in the plastomes and mitogenomes of the 7 *Rosa* species. The results show that the ENC values for the plastomes range from 47.98 to 48.03, while those for the mitochondrial range from 53.54 to 55.61 (**Figure 3**; **Supplementary Table 4**). The plotted data indicate that the ENC values fall below the standard curve rather than aligning with it, suggesting that the PCGs of these species exhibit low codon preference. This implies that the selective pressure on codon usage in the plastomes of *Rosa* species is greater than the effects of mutation. The smaller variation in codon usage among the plastome PCGs compared to the mitogenome PCGs, indicating that plastomes of *Rosa* more likely to use similar codons than the mitogenome.

### 2.4 Mitochondrial structure analysis and gene transfer

**Figure 4:**
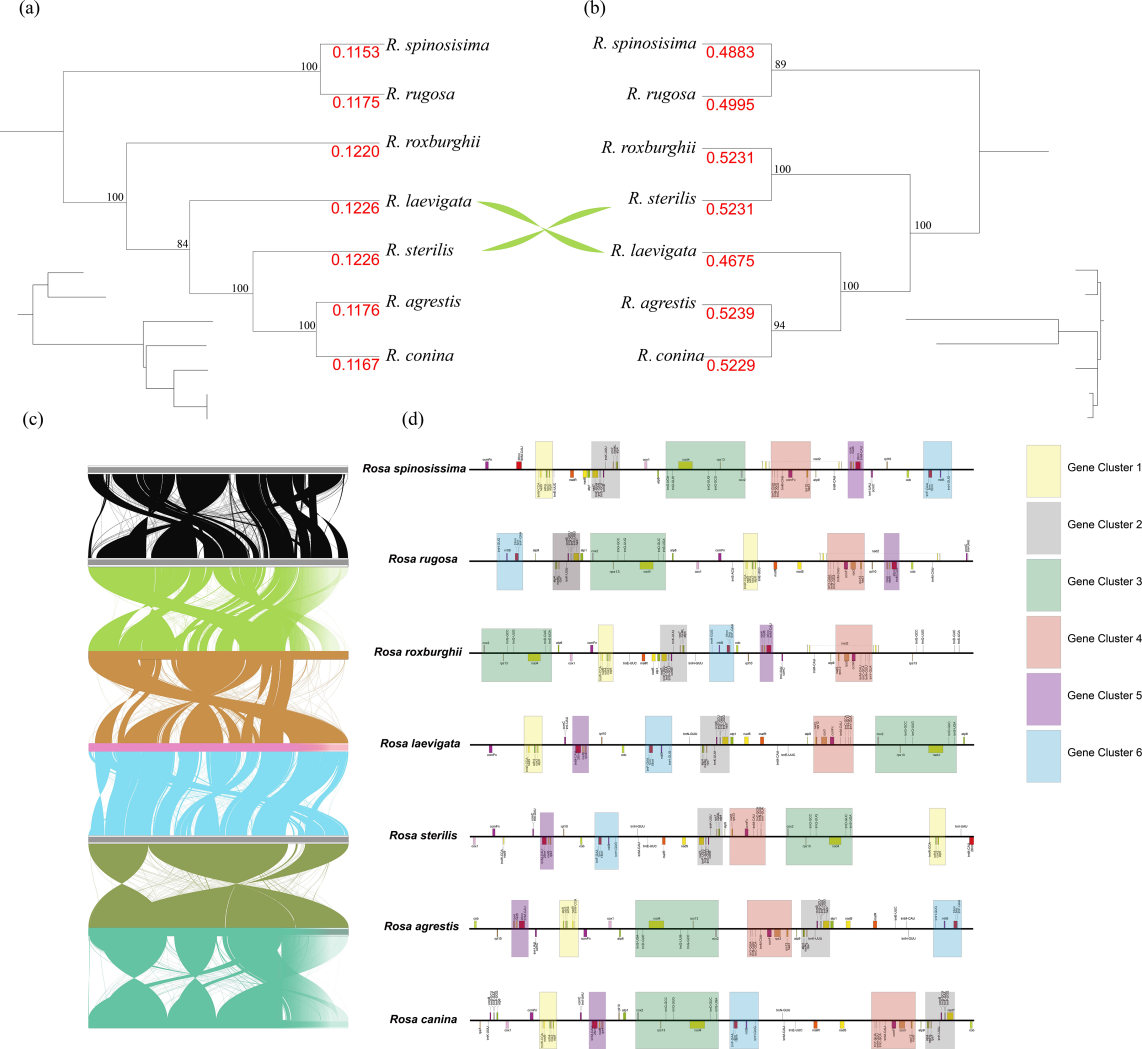
Comparative analysis of organelle genomes. **(a)** The phylogenetic tree based on 78 pt-PCGs. **(b)** The phylogenetic tree based on 31 mt-PCGs. **(c)** Collinearity regions of mitogenomes among 7 *Rosa* species, and **(d)** conserved gene blocks in the mitogenomes of 7 *Rosa* species.

Co-linearity block analysis is a common method for identifying evolutionary relationships among closely related species at the genomic level. In this study, we conducted a co-linearity block analysis of 7 *Rosa* species to investigate structural differences within the mitogenomes of the genus. Our results indicated that, compared to plastomes, the mitogenomes of the 7 species exhibit a more complex co-linear structure (**Figure 4c**). By examining the positions of mitochondrial homologous genes among the 7 *Rosa* species, we found that structural rearrangements within the mitogenomes disrupted gene clusters (**Figure 4d**). Nevertheless, some gene clusters remain conserved across the *Rosa* species, including 6 larger gene clusters (**Figure 4d**). The composition of these clusters is as follows: Cluster 1 consists of *trnW-CCA-nad9-sdh4-cox3-atp8*; Cluster 2 includes *nad7-trnP-UGG-trnF-GAA-trnS-GCU-ccmB*; Cluster 3 comprises *trnS-UGA-trnD-GUC-nad4-trnQ-UUG-trnG-GCC-rps13-cox2*; Cluster 4 contains *trnY-GUA-trnN-GUU-trnC-GCA-trnM-CAU-ccmFc-rps12-nad3*; Cluster 5 is made up of *rps4-nad6-rrn26-trnM-CAU*; and Cluster 6 consists of *trnF-GAA-rrn5-rrn18-mttB-trnH-GUG*.

**Figure 5:**
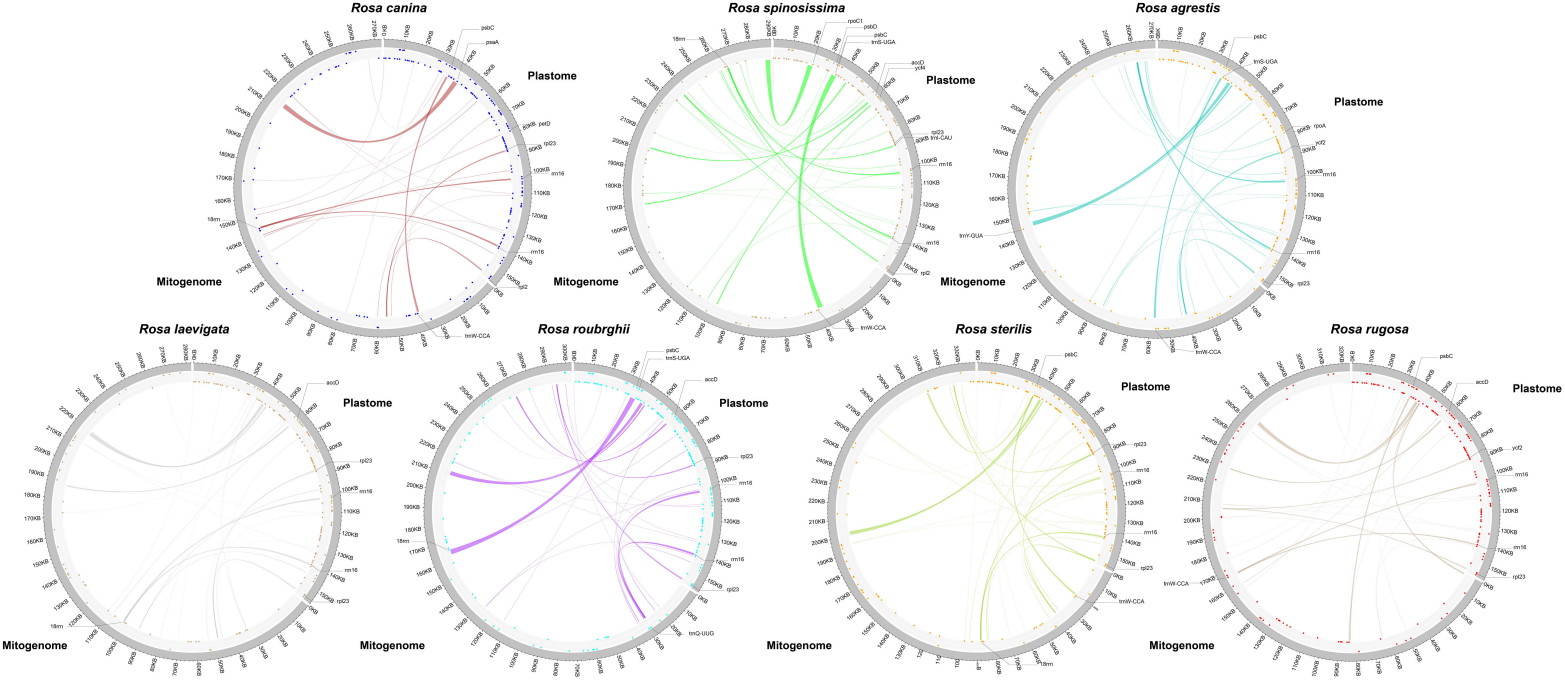
Homologous sequences between the plastome and the mitogenome. In the circle diagram, dots in the inner circle represent genes in the counterclockwise direction and dots in the outer circle represent genes in the clockwise direction. Shaded links represent identified homologous sequences. In homologous sequences complete PCGs are marked with a dash.

Previous studies have reported the presence of plastome gene remnants in the mitogenome, indicating significant sequence transfer between these two organelles. To investigate this, we performed a homology comparison between the plastomes and mitogenomes of the 7 *Rosa* species, illustrated in **Figure 5**. Our analysis detected numerous fragments ranging from 22 to 36 in number, with total lengths spanning from 7,971 bp to 12,132 bp (**Supplementary Table 5**). Notably, *R.* sp*inosissima* exhibited the longest fragments, while *R. canina* had the shortest. Among these fragments, we functionally annotated a total of 14 intact-plastid genes, including 11 PCGs (*accD*, *psbA*, *psbC*, *psbD*, *petD*, *rpl2*, *rpl23*, *rpoA*, *rpoC1*, *ycf2*, *ycf4*), 2 tRNA genes (*trnI-CAU*, *trnS-UGA*), and 1 rRNA gene (*rrn16*) (**Figure 5**). Importantly, we detected that the *rrn16* and *rpl23* genes are part of the intact plastid genes across all 7 species’ plastomes and mitogenomes.

### 2.5 The substitution rates of pt- and mt-PCGs

**Figure 6:**
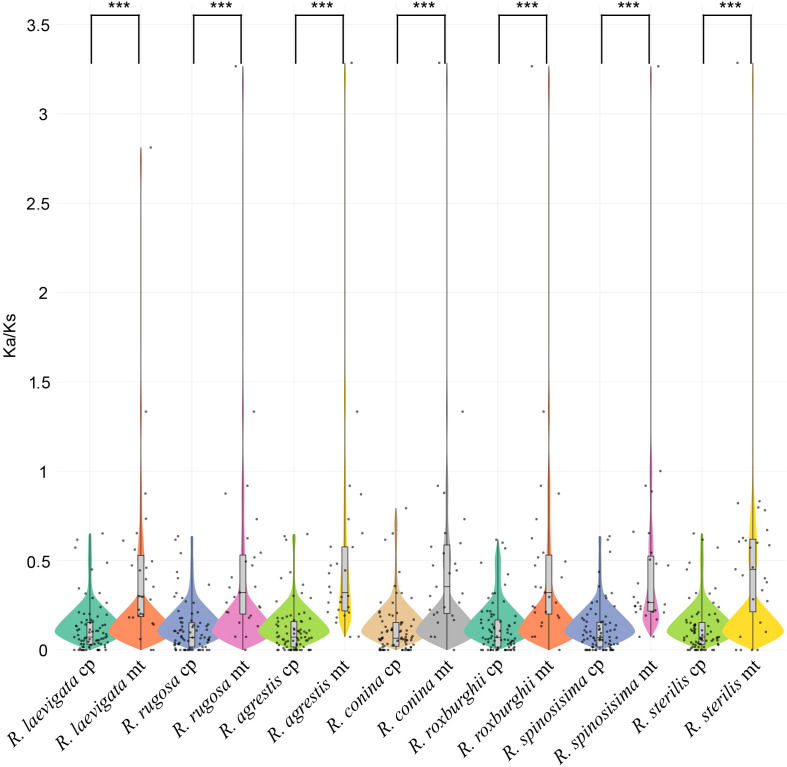
Ka/Ks ratios in mt-PCGs and pt-PCGs of 7 *Rosa* species. ***represents p-value < 0.001.

We assessed the Ka/Ks values of the plastomes and mitogenomes in species of the genus *Rosa*. Our findings revealed significant differences in the Ka and Ks ratios between the two organelles, with average values for mt-PCG ranging from 0.4675 to 0.5231, while for pt-PCG ranging from 0.1115 to 0.1226 (**Figure 6**; **Supplementary Table 6**). Most of the PCGs showed Ka/Ks ratios below 1, indicating that purifying selection has been the dominant evolutionary force acting on these genes. Notably, genes such as *nad4L* and *sdh4* from the mitogenomes exhibited Ka/Ks ratios exceeding 1, suggesting they may have undergone positive selection. The results of the PAML software also supported that these two genes also suffered positive selection in the *Rosa* species (**Supplementary Table 7**). This pattern of differential selective pressure across organelles provides valuable insights into the evolutionary dynamics of plastome and mitogenome in *Rosa* species.

### 2.6 Phylogenetic analysis and divergence time estimation

**Figure 7:**
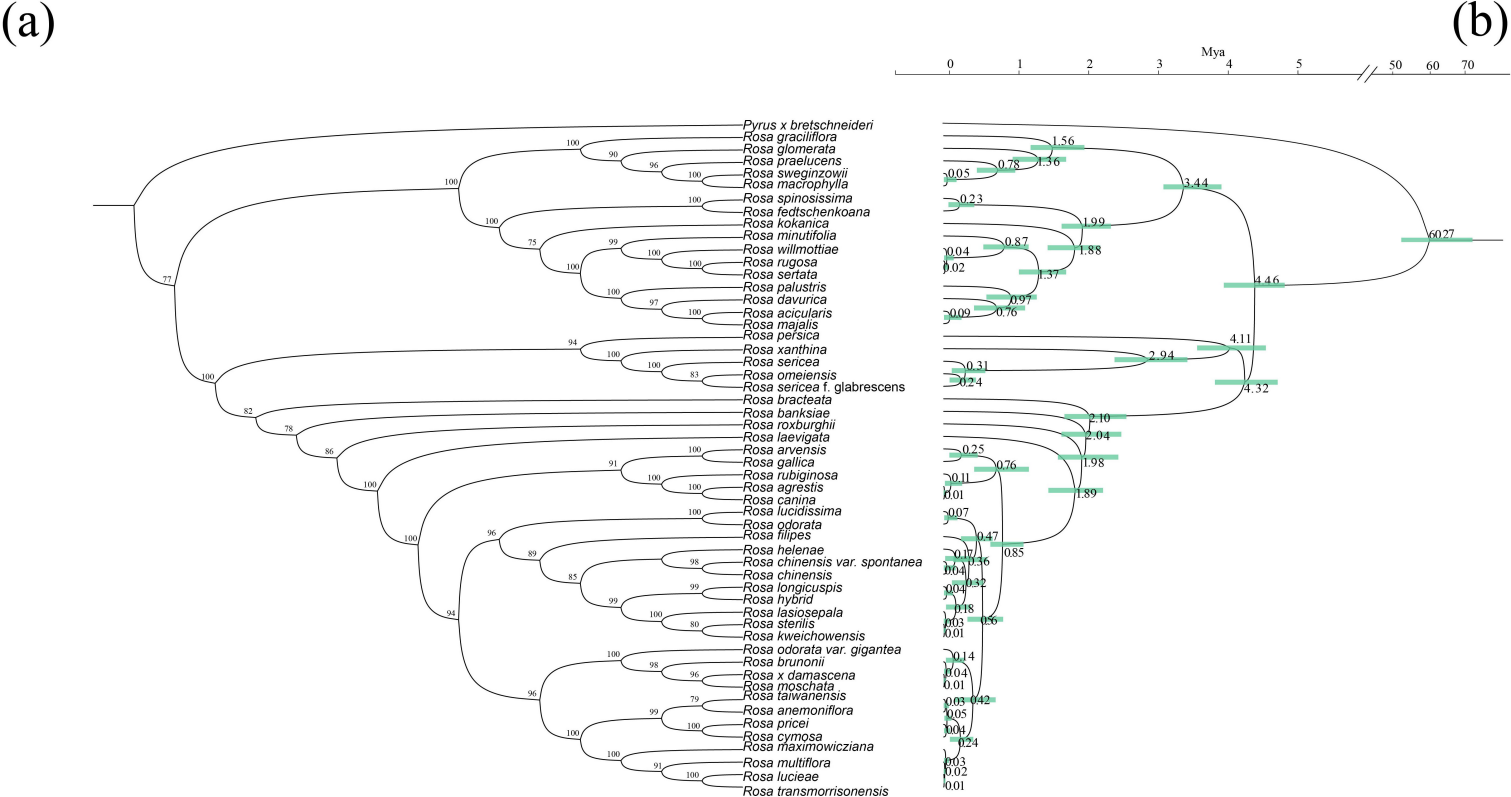
Phylogenetic tree of 7 *Rosa* species. **(a)** The phylogenetic tree based on 78 pt-PCGs. **(b)** Estimates of divergence time of *Rosa* species inferred from an analysis of 78 complete plastomes using Bayesian MCMCTree with the independent-rates relaxed-clock model. Values are shown next to nodes with mean estimates and 95% confidence intervals.

Using 78 PCGs of plastome from 53 *Rosa* species and one outgroup, we constructed a phylogenetic tree with the optimal amino acid substitution model being GTR+F+G4. The phylogenetic results showed strong support, with most nodes having a bootstrap support of 100 (**Figure 7a**). However, the topologies of the phylogenetic trees based on mt-PCGs and pt-PCGs exhibited slight differences (**Figures 4a, b**). The mitochondrial tree strongly supported that *R. sterilis* is sister to *R. roxburghii* (BS = 100; **Figure 4b**). In contrast, the plastid analysis indicated a closer relationship between *R. sterilis*, *R. laevigata*, *R. agrestis*, and *R. conina* (BS = 100; **Figure 4a**).

We estimated divergence times using the mcmctree program based on the plastome maximum likelihood tree, with *Pyrus bretschneideri* as the root species, resulting in a divergence time of ‘>49.2 Mya <77.1 Mya’. This suggested that species in the *Rosa* genus diverged from *Pyrus* around 60.77 Mya. The first divergence within *Rosa* occurred approximately 4.46 Mya. *R.* sp*inosissima* and *R. fedtschenkoana* diverged around 0.23 Mya, while *R. roxburghii* diverged from other *Rosa* species approximately 1.98 Mya. *R. laevigata* diverged from other *Rosa* species around 1.89 Mya, and *R. agrestis* and *R. canina* diverged approximately 0.01 Mya. Finally, *R. sterilis* diverged from *R. kweichowensis* around 0.01 Mya (**Figure 7b**).

## 3 Discussion

In this study, we assembled and annotated the mitogenomes and plastomes of 6 *Rosa* species. Consistent with the typical organellar genome structure of *Rosa* (Jeon and Kim, 2019; Lin et al., 2019; Sun et al., 2020), the plastomes of the 7 *Rosa* species are all circular, ranging in size from 156,342 bp to 157,214 bp. There are slight variations in gene counts among the species, which range from 129 to 131. The mitogenomes are also circular, but they exhibit greater size variation, ranging from 271,191 bp to 338,975 bp, with gene counts varying from 52 to 59. All 7 species possess three rRNA genes in their mitogenomes, aligning with the observation that most land plants have three rRNA genes (Archibald, 2011; Zhang et al., 2024). Notably, the size of mitogenomes in eukaryotes can vary dramatically, ranging from 6 kb to several tens of Mb (Hikosaka et al., 2010; Sloan et al., 2012). Our results indicate that the mitogenome sizes of the 7 *Rosa* species range from 271,191 bp to 338,875 bp, which is comparable to the size of *R. chinensis* (313,448 bp) (Raymond et al., 2018). Therefore, we hypothesize that a mitogenome size around 300 kb is a common characteristic among *Rosa* species. There was a significant difference between the plastome and mitogenome sizes in *Rosa* species, a phenomenon that is consistent with previous findings (Smith and Keeling, ,P, 2015; Wang et al., 2024a). Frequent recombination events in plant mitochondrial genomes may have integrated a large amount of exogenous DNA during evolution, possibly leading to large differences in plant mitochondrial genome size. Furthermore, GC content significantly influences amino acid composition and the structural integrity of genomes during evolution (Wang et al., 2023; Wang et al., 2024). The GC content is typically stable at 43%-46% in higher plants (Herbert et al., 2016a). Our analysis of GC content in the plastomes and mitogenomes of the 7 *Rosa* species reveals that the plastomes exhibit more stable GC content across different species (**Table 1**), whereas the GC content in mitogenomes shows greater variability (**Table 2**). The GC content of the mitogenome of *Rosa* species is in the range of 45.0%-45.6%, which is similar to that of other angiosperm mitogenomes (Chen et al., 2019a; He et al., 2024). This result supported the conclusion that GC content is highly conserved in higher plants (Chen et al., 2019a).

The rearrangements and horizontal gene transfers (HGT) within organelle genomes have been widely demonstrated to play a crucial role in plant evolution, contributing to gene gain and loss, as well as changes in genome size (Notsu et al., 2002; Cheng et al., 2021; Han et al., 2022; Xu et al., 2023). Genes transferred from the plastome to the mitogenome, known as mitochondrial plastid DNA (mitoplastid DNA, MTPT), often manifest as pseudogenes or non-functional rRNA sequences (Bi et al., 2022). In this study, we identified 22 to 36 fragments across 7 *Rosa* species, with the MTPT of *R.* sp*inosissima* being the longest at 12,132 bp, significantly surpassing that of other *Rosa* species. Among these fragments, we detected 14 genes, including 11 PCGs, 2 tRNA genes, and 1 rRNA gene. Notably, we found that *rpl23* and *rrn16* genes belong to the shared MTPTs across the 7 *Rosa* species, suggesting that the transfer of the *rrn16* and *rpl23* genes likely occurred in their common ancestor. Most lost mitochondrial tRNA genes are usually compensated for by corresponding chloroplast-derived tRNA genes, such as *trnH-GUG*, *trnN-GUU*, *trnS-GGA*, and *trnW-CCA* (Notsu et al., 2002; Sun et al., 2024; Wang et al., 2024a; Wang et al., 2025; Zhang et al., 2025a). Consistent with previous research, we identified two complete tRNA genes in the MTPT, further supporting the notion of frequent tRNA gene transfer from plastome to mitogenome (Han et al., 2022; Xu et al., 2023). HGT is a mechanism of genetic material transfer that has been recognized as a significant driving force in biological evolution (Soucy et al., 2015; Wang et al., 2024). The presence of several chloroplast-derived genes in *Rosa* may enhance the species’ adaptability to environmental changes. For instance, *rpl23* is a gene encoding ribosomal proteins, and studies have shown that seedlings with high expression of RPL23A showed significant increases in fresh weight, root length, proline content, and chlorophyll levels under simulated drought and salt stress (Moin et al., 2017). In summary, previous studies have indicated that tRNA genes are transferred to mitogenome in angiosperms via horizontal gene transfer to maintain their function (Bi et al., 2019). Moreover, Yue et al (Yue et al., 2012). found that annotated homologous pt-genes in the genome play key roles in environmental adaptation. Thus, our findings of intracellular gene transfer between organelle genomes provide deeper insights into the adaptive strategies of *Rosa* species during their evolutionary history.

Due to maternal inheritance, haploid characteristics, and ease of assembly, plant plastomes have been widely used to resolve evolutionary relationships among different lineages (Mwanzia et al., 2020; Wang et al., 2023). In contrast, the complex structure of mitogenomes presents greater challenges in obtaining complete sequences, which limits their application in phylogenetic studies. In this study, the phylogenetic relationships of the major lineages in the plastome are consistent with the APG IV system and previous research (Fougere-Danezan et al., 2015). Notably, the evolutionary trees of mitogenomes and plastomes for the 7 *Rosa* species in this study exhibit conflicts. This discrepancy may be attributed to significant differences in the Ka/Ks values of mitochondrial and plastid PCGs (mt-PCGs and pt-PCGs) among the *Rosa* species (**Figure 6**; p < 0.01). As shown in **Figure 4**, the Ka/Ks values of the pt-PCGs for *R. laevigata* and *R. sterilis* are more similar, while those for the mt-PCGs of *R. roxburghii* and *R. sterilis* are more closely aligned. This may contribute to the observed conflicts between plastid and mitochondrial lineages. Meanwhile, previous studies have found that frequent multiple-nucleotide substitutions in *Fragaria* mitogenomes caused by microinversions misled the phylogenetic trouble (Fan et al., 2022). we examined the results of the comparison of mitochondrial PCGs in all *Rosa* species, especially the *sdh4* and *nad4L* genes that showed positive selection, and found that there was a mechanism for mitochondrial PCGs in the *Rosa* species similar to that of *Fragaria* (**Supplementary Figure S2**). Mann-Whitney U tests indicated that the Ka/Ks values of mt-PCGs were significantly higher than those of pt-PCGs (**Figure 6**). Therefore, we hypothesized that this mechanism leads to significantly lower Ka/Ks values in the pt-PCGs than in the mt-PCGs of *Rosa* species.

We observed that most Ka/Ks values of PCG are below 1, suggesting strong purifying selection has acted on these genes throughout evolutionary history. Notably, certain genes, including *nad4L* and *sdh4* from the mitogenomes, exhibit Ka/Ks ratios exceeding 1, indicating they may have undergone positive selection. Evidence of positive selection on the *sdh4* gene has also been reported in *Scheuchzeria palustris* (Wang et al., 2023). As a part of the succinate dehydrogenase (SDH) complex, SDH plays a critical role in the tricarboxylic acid cycle and contributes to energy metabolism, stress responses, and signal transduction (Lemos et al., 2002). Additionally, research indicates a positive correlation between SDH activity and male reproductive cell function, underscoring the importance of assessing SDH activity to evaluate mitochondrial function in male gametes (Rao and Sharma, 2001; Ruiz-Pesini et al., 2001). In various crops, including radish (Su et al., 1996), cotton (Huang, 2003), and non-heading cabbage (Yuan, 2005), enzymatic cytochemical studies have revealed differences in SDH activity between sterile and maintainable lines, implying that low SDH activity may be linked to pollen sterility. Therefore, the positive selection of the *sdh4* gene could be critical for the activity of male gametes in these species. However, due to the limited number of *Rosa* mitogenomes analyzed (only 7 species), we advise caution in drawing definitive conclusions until a broader dataset of *Rosa* mitogenomes become available.

Estimates of divergence times suggest that speciation within the *Rosa* genus occurred at approximately 4.46 Mya, which aligns with earlier findings regarding *Aconitum* (Zhang et al., 2024). Divergence within the *Aconitum* genus is estimated to have taken place around 7.96 Mya, while divergence within Subg. Aconitum taking place about 4.36 Mya. Recent research based on reconstructions of global sea surface temperatures during the late Miocene indicates that a major global cooling event, known as the late Miocene cooling, was triggered by a decrease in CO_2_ levels by approximately 7 Mya (Tzanova et al., 2015; Herbert et al., 2016b). Concurrently, studies by Chen et al (Chen et al., 2019b). demonstrate that the Tibetan Plateau and the Xining Basin experienced substantial uplift during the late Miocene, accelerating aridification across the Asian interior. Since *Rosa* and *Aconitum* are predominantly distributed in southwestern and southeastern China (**Supplementary Figure S3**), we propose that the combined effects of declining CO_2_ levels and the uplift of the Tibetan Plateau may have driven rapid species divergence in the region around the late Miocene.

## 4 Materials and methods

### 4.1 Plant samples and mitochondrial genome sequencing

Wild-growing *R. laevigata* plant specimens were collected from Gaopo Town, Huaxi County, Guiyang City, Guizhou Province, China (106°37′ N 26°17′ E), and the samples were identified by Prof. Yuemei Zhao from Guizhou Education University. Fresh leaves were sampled and immediately frozen in liquid nitrogen for DNA extraction. Subsequently, the pressed voucher specimens were numbered (collection number: Rla_00978) and deposited at the herbarium of Guizhou Education University, China. Total genomic DNA was isolated from fresh leaves using the CTAB method (Doyle and Doyle, 1987). The extracted genomic DNA was used to construct DNA libraries, which were sequenced separately on the Illumina and PacBio platforms at GrandOmics (Wuhan). Second-generation sequencing was performed using the DNB-SEQ-T7 platform, while third-generation sequencing was conducted on the PacBio Sequel II platform. The second- and third-generation data for the other five *Rosa* species were downloaded to the NCBI database and were used for the subsequent assembly of chloroplast and mitochondrial genomes, with specific information provided in **Supplementary Table 1**.

### 4.2 Assembly and annotation of organelle genomes

For the plastome, raw reads were processed to remove adapter sequences and filter out low-quality reads using BBTools (https://sourceforge.net/projects/bbmap/). The filtered data were then assembled into contigs using the GetOrganelle software (Jin et al., 2020). Subsequently, Pilon software (Walker et al., 2014) was employed to polish the assembly with second-generation sequencing data, correcting single nucleotide errors. The resulting genome was annotated using CPGview software (Liu et al., 2023), and manual corrections to the annotations were performed using Geneious software (Kearse et al., 2012). For the mitogenome, we assembled contigs from third-generation sequencing data using PMAT software (Bi et al., 2024). Similarly, Pilon software (Walker et al., 2014) was used to polish the mitogenome assembly, addressing single nucleotide errors. The results were annotated using Geneious software (Kearse et al., 2012), with *R. rugosa* (NC_065237) as the reference. Finally, the plastomes and mitogenomes were then visualized using OGDraw software (Greiner et al., 2019).

### 4.3 Comparative analysis of plastomes

We conducted a comparative analysis of the complete plastomes of 7 species within the genus *Rosa*. The IRscope online tool was utilized to analyze the junctions between the inverted repeat (IR), small single-copy (SSC), and large single-copy (LSC) regions. Sequence variability among the plastomes of the 7 species was assessed using mVISTA with the LAGAN model (Frazer et al., 2004), using R. rugosa as the reference, to illustrate both inter- and intraspecific variations. We extracted the protein-coding genes (PCGs) and intergenic spacers (IGSs) using Geneious software (Kearse et al., 2012), and alignments were performed with MAFFT using default parameters (Katoh and Standley, 2013). The nucleotide diversity of the PCGs and IGSs was analyzed using DnaSP (Librado and Rozas, 2009), employing a sliding window approach with a step size of 200 bp and a window length of 600 bp. CodonW software (Peden, 2000) was used to estimate the codon usage patterns of the PCGs across the plastomes of the 7 *Rosa* species. The codon usage bias for each species was quantified through relative synonymous codon usage (RSCU) values and effective number of codons (ENC). For visualization of the relationship between ENC and GC3s, refer to Zhang et al (Zhang et al., 2024; Zhang et al., 2025a; Zhang et al., 2025b).

### 4.4 Calculation of nucleic acid substitution rates

To compare the Ka/Ks values between the plastomes and mitogenomes of 7 *Rosa* species, we used *Malus domestica* (plastome: MK434916; mitogenome: NC_018554) as a reference for calculating the Ka/Ks values of PCGs. We extracted the coding sequences (CDS) of 77 chloroplast PCGs and 27 mitochondrial PCGs using Phylosuite software (Zhang et al., 2020). The nucleotide sequences of each gene pair were compared using KaKs_calculator software (Zhang et al., 2006) to compute the paired Ka/Ks ratios for the 77 plastid and 27 mitochondrial PCGs. We also calculated Ka/Ks values for all organelle protein-coding genes in rosa species using the branch model (based on phylogenetic relationships) in PAML software (Yang, 2007). Additionally, to compare differences in evolutionary rates between chloroplast and mitochondrial genes, the Ka/Ks values for individual chloroplast protein-coding genes from the 7 mitogenomes were compared and evaluated using a Mann-Whitney U test in R.

### 4.5 Comparative mitogenome analysis and gene transfer

For a comparative analysis of the mitogenome structure of 7 *Rosa* species, regions of covariance between the mitogenomes were identified using Geneious software (Kearse et al., 2012), with ≥80% matches and E-values ≤1e-5. These covariance regions were visualized using the RIdeogram R package (Hao et al., 2020) with default parameters. To identify potential horizontal gene transfers between mitochondria and chloroplasts in 7 *Rosa* species, we used BLASTN software to identify homologous sequences between the organelles (parameters: identity > 80%; e-value=1e-5), and the shinyCircos package was utilized to visualize intracellular gene transfers (IGT) (Chen et al., 2015; Yu et al., 2018).

### 4.6 Phylogenomic analysis

A total of 53 species from the genus *Rosa* and one outgroup species were used for phylogenetic analysis of their plastomes, along with the mitogenomes of 7 *Rosa* species (**Supplementary Table 2**). First, we manually corrected apparent annotation errors in the plastomes of the 54 species and the mitogenomes of the 7 species using Geneious software (Kearse et al., 2012). Subsequently, we extracted the coding sequences (CDS) of 78 PCGs from the plastomes and 31 from the mitogenomes using Phylosuite software (Zhang et al., 2020). The CDS sequences were then aligned using MAFFT software (Katoh and Standley, 2013), and gaps as well as highly variable sites were removed with TrimAl software (Capella-Gutiérrez et al., 2009). We then concatenated the CDS sequences of the 78 chloroplast genes and the 31 mitochondrial genes into a supermatrix (CDS_dataset) using FASconCAT-G software (Kück and Longo, 2014), resulting in a supermatrix with 66,764 chloroplast (cp) and 28,492 mitochondrial (mt) sites for subsequent analyses. The species tree was inferred using the maximum likelihood (ML) method in IQ-TREE software (Nguyen et al., 2015), with 1,000 bootstrap replicates. The optimal nucleotide substitution model was selected using ModelFinder (Kalyaanamoorthy et al., 2017). Meanwhile, in order to explore the phylogeny constructed on the basis of chloroplast and mitochondrial data, phylogenetic analyses were performed based on 7 *Rosa* species for which plastome and mitogenome currently exist. The chloroplast data contained 78 PCGs, the mitochondria contained 31 PCGs, and the models were the optimal alternative models automatically tested and selected by IQ-TREE software (Nguyen et al., 2015). Finally, we visualized the ML tree using ChiPlot (Xie et al., 2023).

The divergence times of *Rosa* species were estimated using the MCMCTree software package from PAML v4.9j (Yang, 2007). Fossil calibration points were obtained from the Timetree5 website (http://www.timetree.org/) and two individual fossil calibration points were selected: divergence times for the genera Pyrus and *Rosa* (23.20 to 29.61 Mya), and divergence times for *R. pricei* and *R. taiwanensis* (2.78 Mya). Finally, the results were visualized using the ChiPlot online website (Xie et al., 2023).

## 5 Conclusions

In this study, we assembled and annotated the mitochondrial genomes of six species from the genus *Rosa* and the plastomes of two species. The chloroplast genome sizes ranged from 156,342 bp (*R. laevigata*) to 157,214 bp (*R. agrestis*), with a maximum difference of 872 bp and a minimum difference of 43 bp. The GC content varied from 37.2% to 37.3%, and the number of genes ranged from 129 to 131. The mitochondrial genomes were all circular, with lengths between 271,191 bp and 338,975 bp, containing 52 to 59 genes. Codon usage analysis indicated a preference for A/T-ending codons in both chloroplast and mitochondrial genes. The ENC-map analysis revealed that the protein-coding genes in both chloroplast and mitochondrial genomes fell below the expected ENC curve, suggesting that mutation plays a minor role in shaping codon preferences. The expansion and contraction analysis of the inverted repeat (IR) regions showed higher conservation in the IR regions compared to the large single copy (LSC) and small single copy (SSC) regions. Additionally, we identified four highly differentiated regions (*rps19*, *ndhF*, *ycf1*, and *psbM-trnD-GUC*) in the plastomes of the 7 *Rosa* species, which can serve as molecular markers for future species identification and studies of genetic diversity.

In contrast to the chloroplasts, the mitochondrial genomes exhibited more complex collinearity relationships, with structural rearrangements disrupting gene clusters. However, some gene clusters remained conserved within the *Rosa* species. Furthermore, compared to PCGs of plastome, mitochondrial PCGs displayed a higher non-synonymous to synonymous ratio. This phenomenon may be due to frequent polynucleotide mutations mediated by microinversions mechanism. We also observed extensive gene transfer between the mitochondria and chloroplasts, particularly with the *rrn16* and *rpl23* genes, which are commonly found in *Rosa* species. These gene transfer events likely occurred in the ancestor of *Rosa* around 4.46 Mya. Estimates of divergence events indicate that rapid differentiation among *Rosa* species took place around 4.46 Mya, potentially influenced by the uplift of the Qinghai-Tibet Plateau during the Late Miocene. This study enriches the genetic resources of the *Rosa* genus and lays the groundwork for the development of molecular markers, phylogenetic analyses, and research into the evolution of organelle genomes.

## Data Availability

The datasets presented in this study can be found in online repositories. The names of the repository/repositories and accession number(s) can be found in the article/**Supplementary Material**.

## Supplementary material

Supplementary Figure 1 Comparison of 7 plastomes using R. rugosa annotation as a reference. The vertical scale indicates the percentage of identity, ranging from 50 to 100%. The horizontal axis indicates the coordinates within the plastome. Genome regions are color-coded as exons, introns and conserved non-coding sequences (CNS).

Supplementary Figure 2 Multinucleotide substitutions and mutation spectra. **(a)** Examples of multinucleotide mutations (MNMs) in Fragaria species (Fan et al., 2022). **(b)** MNMs in sdh4 gene of Rosa species. **(c)** MNMs in nadL4 gene of Rosa species.

Supplementary Figure 3 Global distribution of the two Rosaspecies in this study.

Supplementary Table 1 Source of SRA data used in this study.

Supplementary Table 2 Source of organelle genome files downloaded to the NCBI database in this study.

Supplementary Table 3 Statistics of RSCU values for PCGs in organelle genome from 7 *Rosa* species.

Supplementary Table 4 Summary of GC3s and ENC results for the organelle genomes of 7 *Rosa* species.

Supplementary Table 5 Results of a homology comparison between plastome and mitogenome from 7 *Rosa*species.

Supplementary Table 6 Ka/Ks for the protein-coding genes of the 7 *Rosa* organelle genomes.

Supplementary Table 7 Ka/Ks for the protein-coding genes of the 7 *Rosa* organelle genomes based PAML software.
