# Supplementary figures and images for: Comparative analysis of the organelle genomes of seven Rosa species (Rosaceae): insights into structural variation and phylogenetic position
Source: Front Plant Sci. 2025 May 8;16:1584289. doi: 10.3389/fpls.2025.1584289 (PMC12095378; doi:10.3389/fpls.2025.1584289)

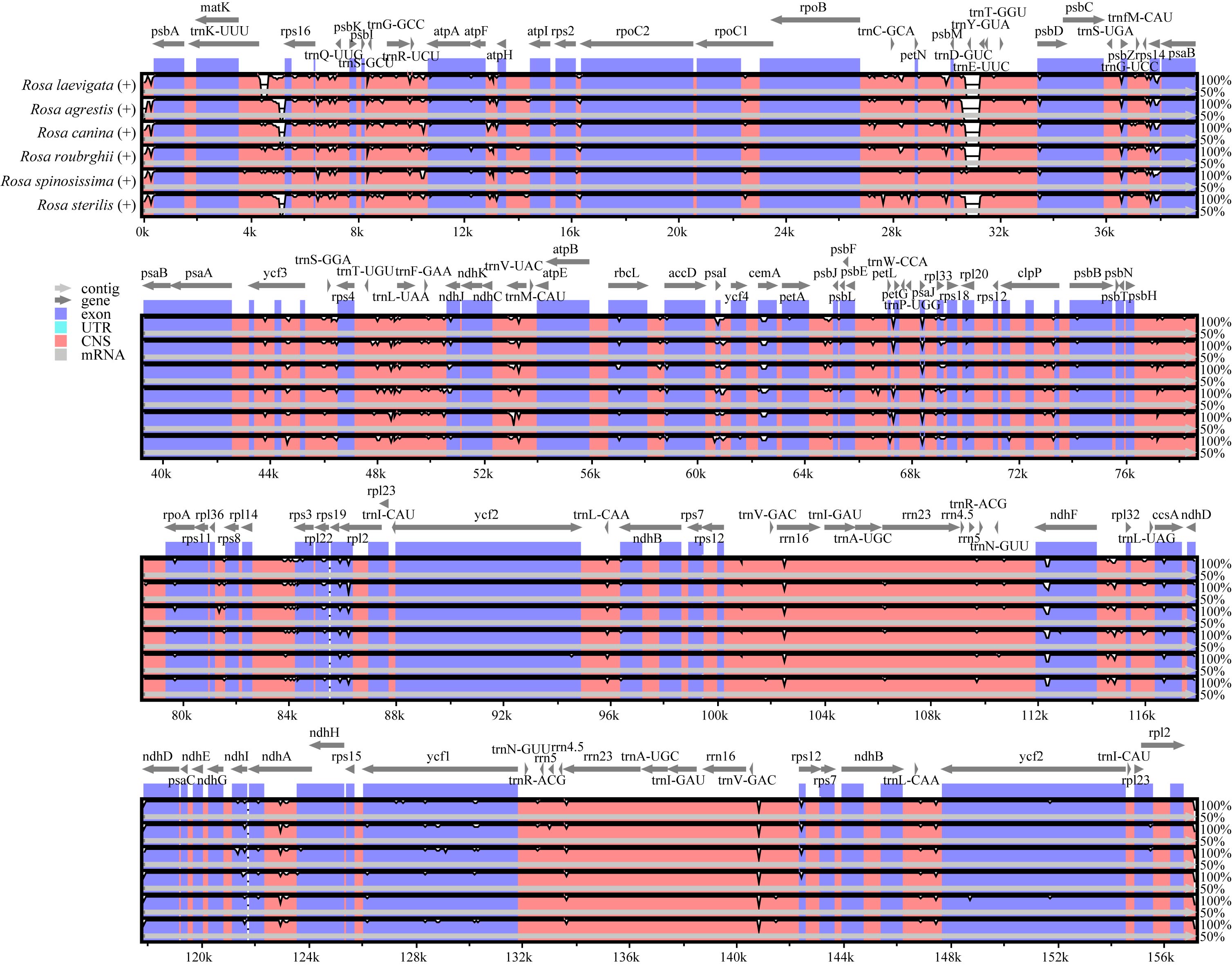

Supplement: Supplementary Figure 1 — Comparison of 7 plastomes using R. rugosa annotation as a reference. The vertical scale indicates the percentage of identity, ranging from 50 to 100%. The horizontal axis indicates the coordinates within the plastome. Genome regions are color-coded as exons, introns and conserved non-coding sequences (CNS). [file Image1.jpg]

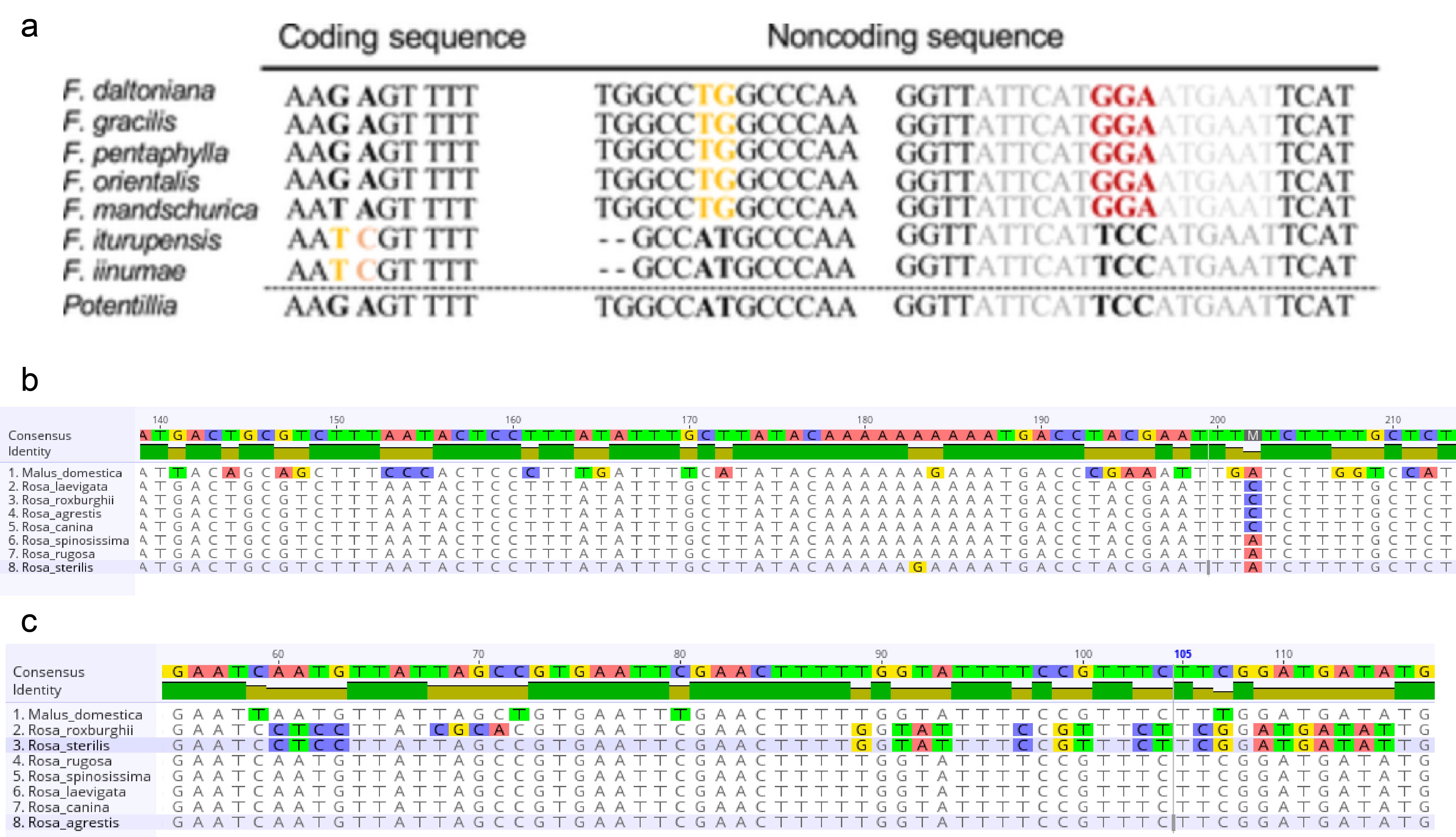

Supplement: Supplementary Figure 2 — Multinucleotide substitutions and mutation spectra. (a) Examples of multinucleotide mutations (MNMs) in Fragaria species (Fan et al., 2022). (b) MNMs in sdh4 gene of Rosa species. (c) MNMs in nadL4 gene of Rosa species. [file Image2.jpg]
